# Introductory

**Published:** 1845-04

**Authors:** 


### ILLINOIS

### MEDICAL & SURGICAL JOURNAL.

VOL. II. APRIL 1845. NO. 1.

THE present number of the ILLINOIS MEDICAL AND SURGICAL JOURNAL commences its second year, and second volume. In the announcement of our publication in the first number of the preceding volume, we acknowledged some doubts of our success. We are happy to say that our doubts are now fully dispelled. Our journal has met with a success which, while it is gratifying to us, establishes it permanently on the list of Medical periodicals. There is but one respect in which we have reason for complaint and regret, viz: that our professional readers have aided us so little by their contributions. We hope that this will be amended. We again extend our invitation to practitioners to contribute to our pages. One of the avowed objects of our journal is to make the medical men of the North-West known to each other; and as ours is still the only medium of communication with the medical world in this vast region of the North-West, we feel that we have a strong claim upon the profession to support our enterprise.

In the department of our journal devoted to Practical Medicine, our exchange list affords us every facility for keeping our readers well apprised, and at an early date, of all improvements of importance in medical practice. In our Bibliographical Notices, we shall, as far as practicable, inform our readers of the issue and relative value of new publications, expressing our opinions with freedom and impartiality. We shall also endeavor to select the most important and spirited portions of Medical Reviews of works which may not be accessible to our readers or ourselves; by this means obviating, as far as practicable, the disadvantages arising from want of access to the large libraries of eastern cities. We will also continue to keep our readers informed of the various topics of interest to the profession, embraced under the head of Medical Intelligence.

The Advertising sheet of our cover is available upon moderate terms, to Druggists, Booksellers, cards of Physicians, Surgical Instrument Makers, &c.

We hope that this, our second volume, will meet with equal favor from our subscribers and contemporaries, with our first. We cannot but think that we have somewhat more experience, and do not intend to relax in the exertions which have sustained our journal in their good opinion.

